# Predicting outcomes in patients with perforated gastroduodenal ulcers: artificial neural network modelling indicates a highly complex disease

**DOI:** 10.1007/s00068-014-0417-4

**Published:** 2014-06-14

**Authors:** K. Søreide, K. Thorsen, J. A. Søreide

**Affiliations:** 1 Department of Gastrointestinal Surgery, Stavanger University Hospital, P.O. Box 8100, 4068 Stavanger, Norway; 2 Department of Clinical Medicine, University of Bergen, Bergen, Norway

**Keywords:** Peptic ulcer perforation, Gastroduodenal ulcers, Mortality, Prediction, Prognosis, Outcome assessment, Computer simulation

## Abstract

**Purpose:**

Mortality prediction models for patients with perforated peptic ulcer (PPU) have not yielded consistent or highly accurate results. Given the complex nature of this disease, which has many non-linear associations with outcomes, we explored artificial neural networks (ANNs) to predict the complex interactions between the risk factors of PPU and death among patients with this condition.

**Methods:**

ANN modelling using a standard feed-forward, back-propagation neural network with three layers (i.e., an input layer, a hidden layer and an output layer) was used to predict the 30-day mortality of consecutive patients from a population-based cohort undergoing surgery for PPU. A receiver-operating characteristic (ROC) analysis was used to assess model accuracy.

**Results:**

Of the 172 patients, 168 had their data included in the model; the data of 117 (70 %) were used for the training set, and the data of 51 (39 %) were used for the test set. The accuracy, as evaluated by area under the ROC curve (AUC), was best for an inclusive, multifactorial ANN model (AUC 0.90, 95 % CIs 0.85–0.95; *p* < 0.001). This model outperformed standard predictive scores, including Boey and PULP. The importance of each variable decreased as the number of factors included in the ANN model increased.

**Conclusions:**

The prediction of death was most accurate when using an ANN model with several univariate influences on the outcome. This finding demonstrates that PPU is a highly complex disease for which clinical prognoses are likely difficult. The incorporation of computerised learning systems might enhance clinical judgments to improve decision making and outcome prediction.

## Introduction

Perforated peptic ulcers (PPUs) are the leading cause of surgery-related death worldwide [1]. Although the incidence of peptic ulcer complications due to bleeding has decreased [2], the incidence of perforations has remained stable over the past few decades despite surgical and medical advancements. PPU is a severe complication of peptic ulcer disease with a reported mortality of approximately 10–20 %, even in modern surgical series [3–7].

Mortality prediction is of importance, but previous models have yielded inconsistent results [8, 9]. Further, the Boey score [10] as one of the most frequently used scores was created on patient series during the 1980s, which may explain why results vary across studies [9]. In fact, we recently demonstrated that no predictive models has superior accuracy [11]; at best, only 4 out of 5 patients are correctly classified by any particular model.

Notably, although prediction was improved by a previous model that combined six pre-operatively obtainable variables [11], the prognostic value of any single factor was limited. This limitation might be explained by the fact that biological systems have relationships between the variables that are complex, multidimensional and non-linear.

The increasing availability of electronic medical information that can be collected and used for pattern recognition has created new opportunities to improve diagnoses and predictions of disease outcomes [12–15]. Computers can gather and process thousands of variables as well as learn to recognise patterns by simulated “trial-and-error” processing—often referred to as “artificial intelligence”. One such type of artificial intelligence is the artificial neural network (ANN). ANNs are information-processing paradigms inspired by the analytical processes of the human brain. Emerging data have demonstrated the superiority of ANN modelling with regard to several benign or malignant gastrointestinal disorders [16–19]; however, ANNs have never been applied to predict PPU outcomes. Thus, our objective was to explore the ability of an ANNto improve survival prediction.

## Materials and methods

The study was approved as a quality control assurance project according to the Regional Ethics Committee (REK Vest # 2011/713). The study complied with the Strengthening the Reporting of Observational Studies in Epidemiology (STROBE) statement, where applicable [20].

The study cohort has been described in detail elsewhere [11]. A population-based consecutive series of 172 patients diagnosed and operated on for perforated gastroduodenal ulcer between January 2001 and December 2010 were included in the current study. Patients with perforations caused by malignant disease (i.e., gastric cancer) and patients who did not undergo surgery were excluded from the current study. The primary endpoint of this study was the 30-day mortality after surgery for PPU.

The clinical and laboratory variables have been defined previously [11]. Optimal cut-off values were established using ROC analysis for dichotomising continuous variables, as described elsewhere [21]. Both the Boey score [22] and the PULP score [23] have been described elsewhere.

### Predicted probabilities from regression analyses

A multivariate regression analysis was performed as previously described [11] to evaluate the current PPU scoring systems, including Boey and PULP. For the regression models, the saved probabilities for each patient in the model (either Boey or PULP) were used for comparison with the output values generated for ANN modelling in the current study.

In the previous multivariate regression model that used the same material [11], mortality was best predicted based on a combination of negative prognostic factors: increasing age, the presence of an active cancer, a delay from admission to surgery >24 h, hypoalbuminaemia, hyperbilirubinaemia and increasing creatinine values. The predictive probabilities for the multivariate model had an AUC of 0.89.

### Statistical analyses

The data were analysed using the Statistical Package for Social Sciences (IBM SPSS v. 21, Inc. for Mac).

The ANN model used in the current study was a multilayer perceptron (MLP) network conducted as a standard feed-forward, back-propagation neural network with three layers: an input layer, a hidden layer and an output layer. The MLP network is a tool for designing special classes of layered feed-forward networks. The input layer consists of source nodes, and its output layer consists of neurons; these layers connect the network to the outside world. In addition to these layers, the MLP usually has one or more layers of neurons referred to as hidden neurons because they are not directly accessible. The hidden neurons extract important features contained in the input data.

The patients were randomly divided into a training/cross-validation group (70 %) and an internal validation group (30 %). The training/cross-validation group was used to train the network.

We initially constructed the present MLP network with six input neurons derived from the previous logistic regression model, which included only objective and reproducible laboratory variables (i.e., age, surgical delay, the presence of active cancer and blood laboratory values for serum albumin, bilirubin and creatinine).

### Definitions of the ANN models

**Fig. 1:**
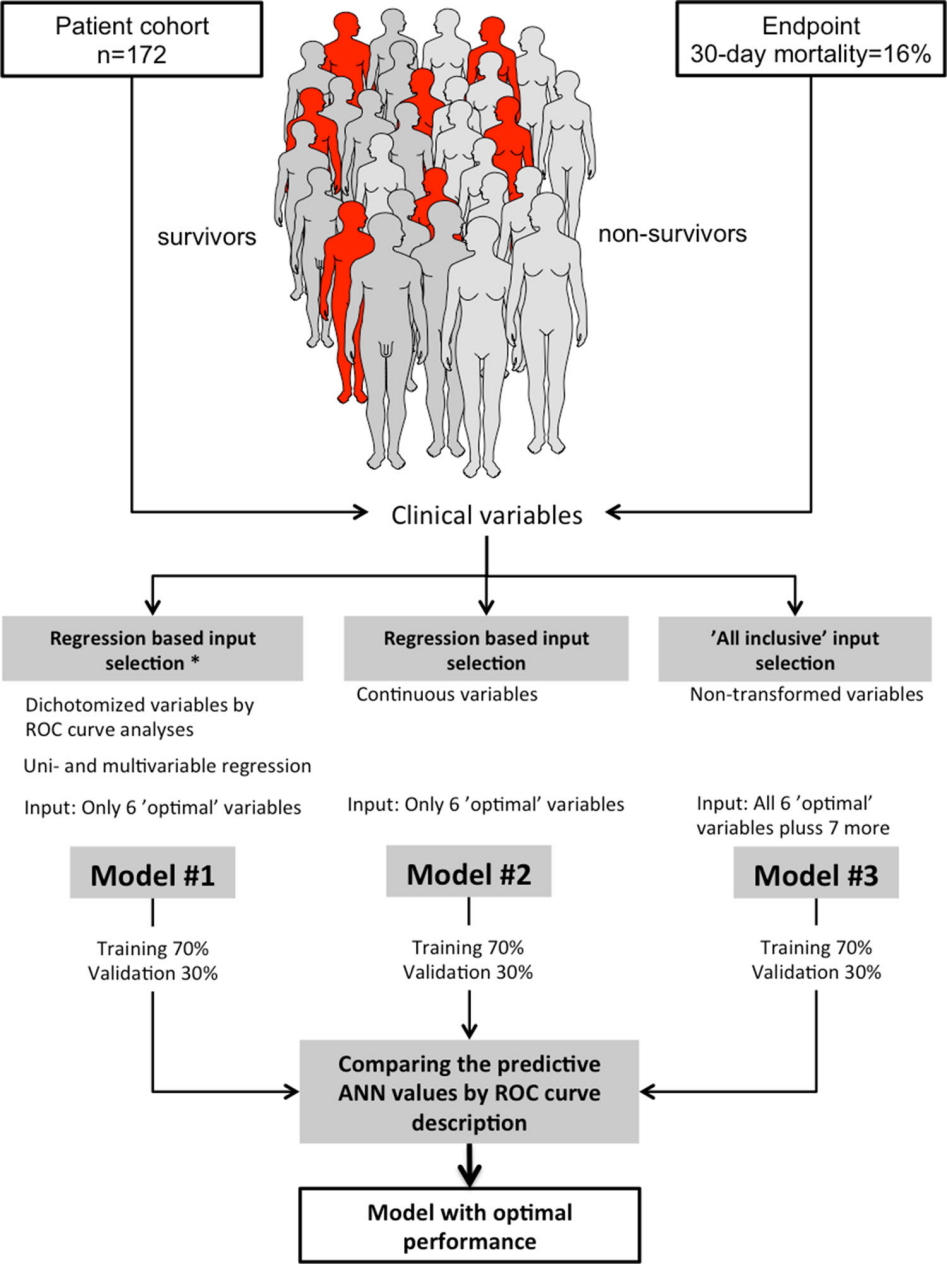
Flow-chart of experiment for each model. *Asterisk* regression based on variables selected in multivariable analyses from Thorsen et al. [11]

The experimental design is visualised in Fig. 1. The first model (hereafter referred to as model #1) was created using the same input variables as previously established in uni- and multivariate regression analyses [11], with dichotomised variables for continuous data based on optimal cut-off values in the ROC analysis (e.g., to indicate hypoalbuminaemia, hyperbilirubinaemia and increased creatinine values; Table 1). In the second model (model #2), the same variables were included but used as non-dichotomised, continuous values to allow the model to adjust the role of each variable throughout the spectrum of values.

**Table 1:** Variables included in the neural network modelling

Factors (units)	ANN mod. #1	ANN mod. #2	ANN mod. #3	Boey	PULP
Gender (M/F)			**✔**		
Age (years)	✔	✔	✔		✔
Location of ulcer (duodenal/gastric)			✔		
Diagnostic delay (h)					✔
Delay before surgery (h)	✔	✔	✔	✔	
Type of surgical repair (lap/open)			✔		
Comorbidity (any)				✔	
ASA fitness score (I–V)			✔		✔
Active cancer disease (y/n)	✔	✔	✔		✔
Liver cirrhosis (y/n)					✔
Steroid use (y/n)					✔
Albumin (g/L)	✔	✔	✔		
Bilirubin (μmol/L)	✔	✔	✔		
Creatinine (μmol/L)	✔	✔	✔		✔
Leucocytes (10^9^/L)			✔		
C-reactive protein (mg/L)			✔		
Sepsis on admission (y/n)			✔		
Shock on admission (y/n)			✔	✔	✔

For comparison, the variables included in the Boey and PULP scores are shown

Finally, a third model (model #3) was created to explore the enhanced capabilities and unknown interactions of variables. This model included all of the potential factors that might be associated with mortality, including gender, the presence of shock at admission, sepsis and mode of surgery.

To compare these three models, the output predictive value for each ANN was compared using ROC analysis and 95 % confidence intervals. All *p* values <0.050 were considered significant.

## Results

**Fig. 2:**
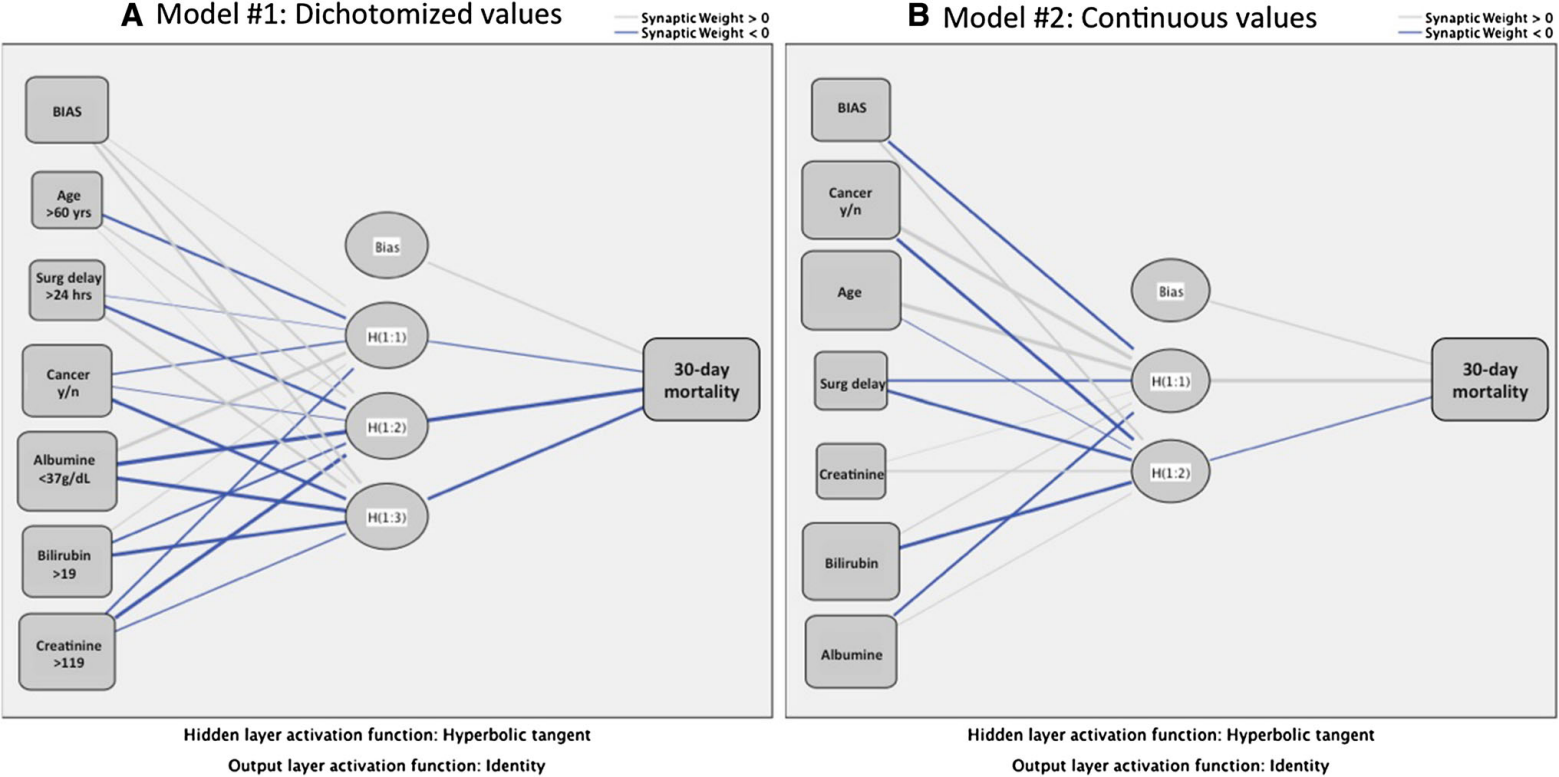
Two ANN models. Although the same input variable nodes were kept, the network and hidden nodes changed when using continuous data (in model #2) versus dichotomised values (in model #1), demonstrating a change in the importance and the relationships between each variable and the outcome

A total of 172 patients were included; 28 deaths occurred within 30 days of surgery (16 %). The baseline data are presented in Table 2. The networks created for ANN models #1 and #2 are shown in Fig. 1. The six input nodes resulted in different hidden layers for outcome predictions when either dichotomised (Fig. 2a) or continuous (Fig. 2b) variables were imputed. Notably, the accuracy of the model was reduced by the latter approach (Table 3).

**Table 2:** Baseline characteristics of the patients with PPU

Characteristics	Total (*n* = 172)	Deaths at 30 days (*n* = 28)
Gender, F:M	89:83	17:11
Age, median years (range)	68 (18–100)	80 (56–95)
Ulcer in prior history (*n*, %)	26 (15 %)	5 (18 %)
Location of ulcer, duodenal:gastric	60:112	14:14
Delay to surgery, median hours (range)	6.2 (0.5–116.2)	10.0 (1.1–40.6)
Laparoscopic repair	50 (29 %)	8 (29 %)
Shock at admission	37 (22 %)	10 (37 %)
Sepsis at admission	70 (42 %)	12 (48 %)
ASA fitness score ≥III	73 (42 %)	24 (86 %)
Active cancer disease	19 (11 %)	9 (32 %)
Boey score ≥2	56 (33 %)	18 (64 %)
Steroid use	16 (10 %)	5 (18 %)

The data are presented as numbers and (%) or as medians with ranges

**Table 3:** ROC analysis with AUC and 95 % CIs for the different ANN models

Test result variable(s)	AUC	AUC 95 % CI	*P* value
Neural network model #1	0.84	0.77–0.91	<0.001
Neural network model #2	0.77	0.67–0.87	<0.001
Neural network model #3	0.90	0.85–0.95	<0.001

**Fig. 3:**
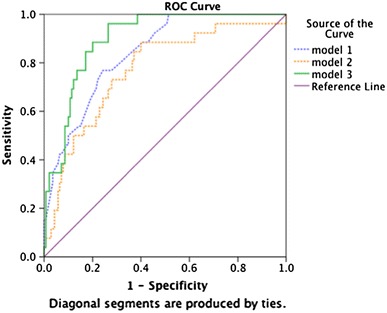
ROC curve comparing the accuracy of the three ANN models

ANN model #1 had an accuracy that was somewhat lower than that of a previously reported regression model. The AUC of the ANN was 0.84, whereas the AUC of the logistic regression model was 0.89 using the same variables [11]. The accuracy of the model decreased (model #2) when the variables were input as continuous measures (Table 3). Increasing the available information in the model by liberally including numerous variables (ANN model #3) with an unknown relationship to the outcome improved the accuracy of the model to AUC 0.90 (Table 3; Fig. 3). In clinical terms, this result means that the model should accurately predict death in 9 out of 10 patients undergoing an operation for PPU.

The constructed graphs for each model in the network analysis suggest that accuracy increased when more variables were added to the model (Fig. 3). When including additional variables such as the presence of comorbidities (e.g., cardiovascular, pulmonary or autoimmune diseases), the accuracy of the model decreased (data not shown), which indicates that additional predictive information was not available.

**Fig. 4:**
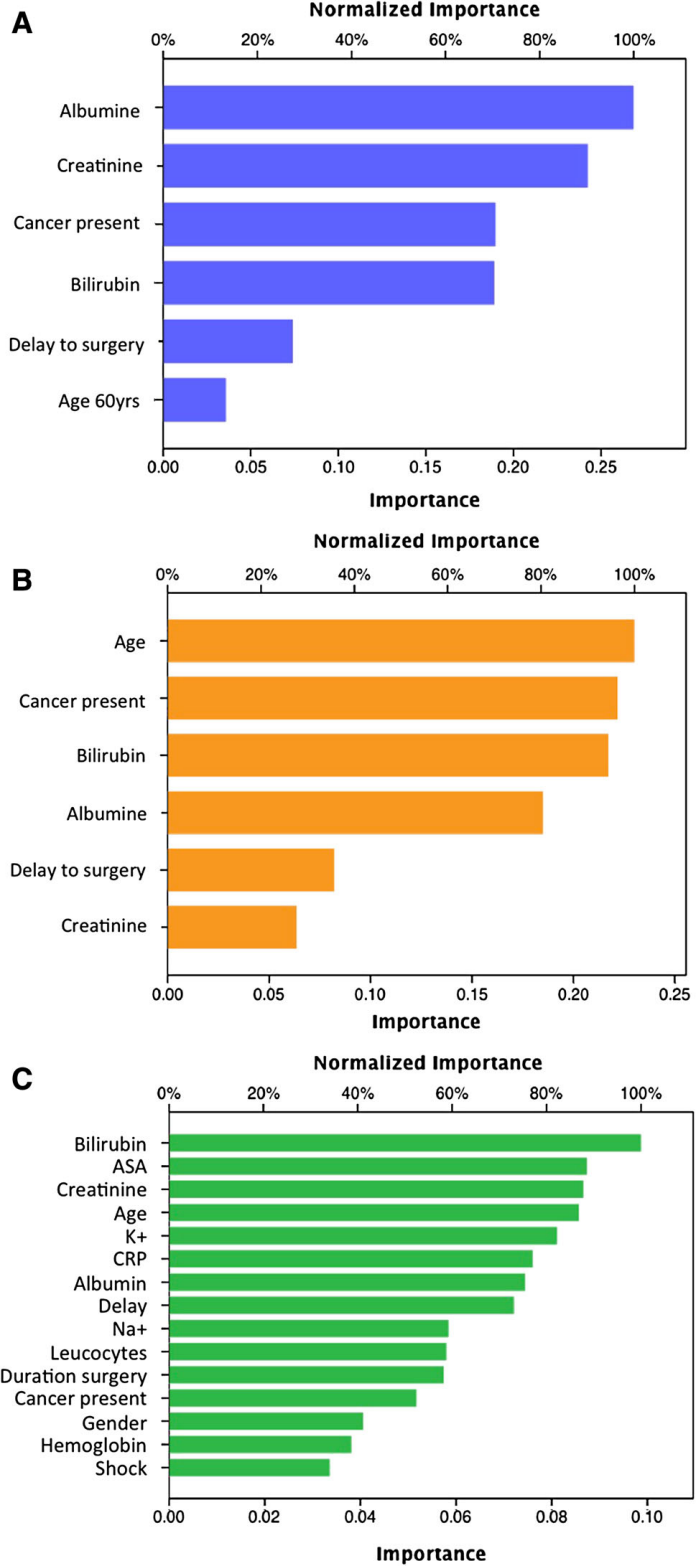
Weighted importance of included variables for each model. Both variable importance and the relative weight (or contribution) of the predictive models shifted, as indicated on the *x*-axis of each graph **a** for model #1; **b** for model #2 and **c** for model #3

Furthermore, as the number of variables in the models increased, the predictive value of each variable decreased considerably (Fig. 4a–c). In fact, the incremental input from each variable was small (Fig. 4c), although the model’s prediction accuracy increased (Fig. 3). In addition, the relative contribution of each variable changed considerably (Fig. 4a–c), as expressed by the variable importance.

The weighted importance of the factors changed; a strong emphasis was placed on CRP, creatinine and bilirubin; however, the other factors (including age, shock, active cancer or other risk factors) exerted virtually no effect.

ANN model #3 was more accurate than a previously reported multivariate regression model based on increasing age, the presence of active cancer, an admission delay for surgery of >24 h, hypoalbuminaemia, hyperbilirubinaemia and increased creatinine values (model AUC of 0.89) [11]. However, the increase in accuracy was not significant (from an AUC of 0.89–0.90), showing an overlap of the 95 % CIs for both models.

## Discussion

This study demonstrated that the application of ANNs to enhance the outcome predictions regarding patients with PPU slightly improved upon the predictive ability of a previously reported regression model [11]. Furthermore, the ANNs performed better than the currently proposed Boey and PULP scores for outcome predictions among patients with PPU. Of note, the ANN models suggested a difference in the weighted importance of the included factors that changed based on how the variables were included and combined in the model. Based on the differences in the model inputs and the resulting outputs, outcome predictions of PPU were complex and likely to be influenced by several factors that might have unknown relationships with each other (which cannot be easily demonstrated using standard near-linear, regression models). The cumulative presence of unknown and small relationships might largely explain the difficulty, limited success and moderate accuracy associated with generating predictive models consisting of a few (e.g., 3–5) explanatory variables, given that the actual outcome depends on a much greater variance and (at least partially unknown) interdependence of factors. Drawing an analogy to the clinical setting, the inexperienced physician may be overwhelmed of the number of factors to consider, some with subtle meaning while others are more predominating, thus preventing the clinician to consider any more than a blunt few for clinical decision making. In reality, many subtle factors may indeed point to a potential dire situation that may easily be missed or go unrecognised by the inexperienced clinician. Situations such as these are where computerised pattern recognition and prediction algorithms can become useful [24]. Although their full potential has yet to be reached [25], developments in technology are rapidly moving toward models that might become available for everyday use. A further example of “innovation technology” might be the rapid and widespread use of information technology such as smart phones and various applications [26] that only a decade ago seemed futuristic and at a developmental stage at best.

ANN-based models might even help expert clinicians. The belief that the brain’s short-term memory can simultaneously retain (and therefore optimally use) only 4–7 pieces of information is of importance; attempts to use larger amounts of information at one time can lead to ineffective decision-making. Given this limitation, many clinicians might have difficulty assimilating the many variables that are often encountered in real-life clinical environments. This mismatch between innate human ability (i.e., “human brain processing capacity”) and excessive input data (i.e., “information overload”) might contribute to unnecessary variations in clinical practice (i.e., “decision-making”), poor compliance with established guidelines and even errors in medical judgment [27]. In fact, certain computer systems are beginning to reveal clinical implications in several areas to improve patient safety and generate complex data analyses [12–14].

Notably, an experienced clinician may outperform the ability of an advanced computer prediction models. However, clinical decision-making is based on the human ability to collect information and process it into clinically predictive patterns. This skill is influenced by knowledge, experience and sources of bias. Knowingly, becoming an “expert” takes thousands of hours of experience—a fortune that clinicians do not have from the start and which may take longer time to require in the current work-restricted environment [28]. The increasing availability of electronic medical information that can be collected and used for pattern recognition has created new opportunities to improve diagnoses and predictions of disease outcomes [12–15]. Computers can gather and process thousands of variables as well as learn to recognise patterns by simulated “trial-and-error” processing, often referred to as “artificial intelligence”. In other words, computers are able to make informed decisions. Such technology is already in use in aviation systems, banking technology, industrial robotics and certain areas of medicine [29, 30]. While we do not suggest that computer systems will replace human input, there may be a source for potential improvement in pattern recognition that the inexperienced human brain is incapable of, at least until obtaining thousands hours of experience. It may indeed represent the “gut feeling” of the experienced clinician that recognise a premonition without being able to pin-point the exact determinator for it.

The potential medical applications of ANNs include scenarios in which the relationship between independent variables and clinical outcomes are poorly understood [31, 32]. Because ANNs are capable of self-training with minimal human intervention, many studies of large epidemiology databases have used ANNs in addition to traditional statistical methods to gain additional insight into the relationships among variables. Several previous studies have used ANNs to predict mortality after surgery [16, 33–35]. ANNs have also been used to aid in the diagnosis and determination of disease severity [17, 19, 36]. ANNs are particularly suited to solving non-linear problems and analysing complex datasets [32]. As such, ANNs constitute potent alternative computational tools that are able to outperform the diagnostic and prognostic accuracy of classical statistical methods.

Of note, ANNs and other artificial intelligence systems are only as smart as we make them. Thus, there will always be a need for human input of what sort of data, the quality of data and the source of data collection that the ANN may utilise. Accordingly, the danger of “garbage-in, garbage out” may exist. Also, most clinicians (and researchers) may be uncomfortable with the “hidden nodes” which are essentially unavailable and intangible elements of the computer process. Also, if input is obtained on a one-time point basis, it may miss the dynamics of process. However, in real-time models that captures and process variables continuously, this limitation may be overcome. One example may be the continuous monitoring performed in intensive care units, for which considerable data amounts may be difficult to assess for the human brain, but may yield threshold values (i.e., express risk of adverse event or further deterioration) in a learning, artificial intelligence system.

The current strict, population-based, non-selected cohort is a particular strength of this study because it reduces transferral bias or other selection criteria that are found in regions with coverage overlap between hospitals. However, all studies have limitations, and the current study is partially limited by its moderately sized sample and reliance on previously defined predictive variables (e.g., the best cut-off values for dichotomous variables) for building the models. A true, secondary and external validation cohort is lacking. If such a cohort were present, this study would have enhanced generalizability, but no such cohort was available when the project began. However, the cohort was split into training and test sets for the modelling. Notably, no selection bias was present with regard to the patients recruited for this study because SUH is the only hospital that provides care for the target population. Thus, the results might have external validation with regard to other Western populations. However, the results might not apply to perforated ulcer outcomes in regions where this disease has a different patient profile, such as Africa. Accordingly, to build on the results from this study, an international cohort of patients from various geographic regions should be sought. In addition, a unified agreement concerning data variables and inclusion is needed because these standards can differ across studies.

Globally, PPU is associated with a major surgical disease burden; however, randomised trials and prospective investigations are few and far between [37]. Additional international collaborations to increase the power of trials to generate more robust results should be pursued to improve care and eventually outcomes [38]. One of the predetermining factors for creating trials or comparing outcomes in PPU management is the possibility of allocating patients to risk based on agreed methods and consistent definitions. If the implementation of ANN is validated in larger and external series, this modelling might prove beneficial in terms of risk stratification or treatment allocation in a prospective setting. Notably, the current ANN model (AUC of 0.90) correctly identified 9 out of 10 patients at risk of dying within 30 days after PPU surgery. Furthermore, the long-standing issue of non-operative treatment and defining the best candidates for this treatment does not currently have acceptable prediction models [39]. Thus, focus should be placed on improving the prediction accuracy to generate reliable and robust models for future risk stratification and potential treatment allocation for patients with PPU.
